# ChatGPT for Univariate Statistics: Validation of AI-Assisted Data Analysis in Healthcare Research

**DOI:** 10.2196/63550

**Published:** 2025-02-07

**Authors:** Michael R Ruta, Tony Gaidici, Chase Irwin, Jonathan Lifshitz

**Affiliations:** 1 University of Arizona College of Medicine – Phoenix Phoenix, AZ United States

**Keywords:** ChatGPT, data analysis, statistics, chatbot, artificial intelligence, biomedical research, programmers, bioinformatics, data processing

## Abstract

**Background:**

ChatGPT, a conversational artificial intelligence developed by OpenAI, has rapidly become an invaluable tool for researchers. With the recent integration of Python code interpretation into the ChatGPT environment, there has been a significant increase in the potential utility of ChatGPT as a research tool, particularly in terms of data analysis applications.

**Objective:**

This study aimed to assess ChatGPT as a data analysis tool and provide researchers with a framework for applying ChatGPT to data management tasks, descriptive statistics, and inferential statistics.

**Methods:**

A subset of the National Inpatient Sample was extracted. Data analysis trials were divided into data processing, categorization, and tabulation, as well as descriptive and inferential statistics. For data processing, categorization, and tabulation assessments, ChatGPT was prompted to reclassify variables, subset variables, and present data, respectively. Descriptive statistics assessments included mean, SD, median, and IQR calculations. Inferential statistics assessments were conducted at varying levels of prompt specificity (“Basic,” “Intermediate,” and “Advanced”). Specific tests included chi-square, Pearson correlation, independent 2-sample *t* test, 1-way ANOVA, Fisher exact, Spearman correlation, Mann-Whitney *U* test, and Kruskal-Wallis *H* test. Outcomes from consecutive prompt-based trials were assessed against expected statistical values calculated in Python (Python Software Foundation), SAS (SAS Institute), and RStudio (Posit PBC).

**Results:**

ChatGPT accurately performed data processing, categorization, and tabulation across all trials. For descriptive statistics, it provided accurate means, SDs, medians, and IQRs across all trials. Inferential statistics accuracy against expected statistical values varied with prompt specificity: 32.5% accuracy for “Basic” prompts, 81.3% for “Intermediate” prompts, and 92.5% for “Advanced” prompts.

**Conclusions:**

ChatGPT shows promise as a tool for exploratory data analysis, particularly for researchers with some statistical knowledge and limited programming expertise. However, its application requires careful prompt construction and human oversight to ensure accuracy. As a supplementary tool, ChatGPT can enhance data analysis efficiency and broaden research accessibility.

## Introduction

ChatGPT is a conversational artificial intelligence (AI) created by OpenAI. It has quickly become an invaluable resource for researchers with capabilities that include reviewing literature, identifying gaps in research, and drafting papers [1-5]. With the recent addition of Python code interpretation to the ChatGPT environment, a surge of new research applications has emerged, particularly in data analysis [6]. Accompanied by innovations such as data upload and download, this new feature marks a considerable advancement toward individualized AI-assisted data analysis.

The accessibility of ChatGPT has the potential to democratize data analysis for nonspecialists and serve as a bridge between programming knowledge and the growing demands of biomedical research [7]. Previously, ChatGPT had proven to be an asset to programmers, with capabilities ranging from debugging and annotating code to translating between coding languages [8-10]. However, for individuals without coding experience, deploying a local programming environment posed a significant challenge [7]. ChatGPT’s ability to interpret code bypasses this barrier of entry, making language-to-code translation more accessible [6].

ChatGPT has excelled in some recent data analysis applications by completing bioinformatics exercises with high accuracy [6]. A preliminary analysis of ChatGPT’s utility as a data analysis tool found that it provided results consistent with traditional biostatistical software [11]. However, the extent to which ChatGPT can assist with data analysis when the research question is cross-disciplinary remains unclear. For example, ChatGPT’s utility was limited in addressing complex bioinformatics tasks, citing restricted file size and exclusive support for Python as key obstacles [7]. Other studies have recommended against using ChatGPT for statistical analysis due to incorrect answers, mislabeled data, and speculative results [12-14]. Continual improvements to the GPT models and refined prompts may improve the value proposition of ChatGPT in biostatistics.

As the capabilities of AI technology expand, the next step in democratizing research is to develop ChatGPT’s applications for basic statistical analysis. Our research aims to provide a framework for performing preliminary inferential and descriptive statistics, as well as data management within ChatGPT. The ultimate goal is to avail a more equitable research landscape without the barriers presented by coding knowledge, thereby broadening access and innovation within the research community.

## Methods

### Dataset and Analyses

The 2019 National Inpatient Sample data from the National Healthcare Cost and Utilization Project was selected to assess ChatGPT’s capabilities. This dataset was chosen to represent real-world observational data, similar to typical clinical research studies. Also, these data require processing before testing an array of hypotheses with various statistical tests. This is not a public-use dataset. For researchers interested in replicating these methods, this dataset is often available through institutional access.

Arbitrary inclusion criteria were selected to make the data both manageable and relevant to the aims of this study. ChatGPT’s data and processing limits required uploaded files to be less than 100 megabytes. The inclusion criteria were individuals aged 41-70 years old who endured both a cerebral infarct and a myocardial infarction, with total hospital charges less than US $400,000. The resulting dataset included 2740 observations each with 5 attributes: age, gender, race, length of stay, and total charges. The 5 attributes included continuous, categorical, and binary data elements.

Common statistical methods for univariate analysis were used to evaluate ChatGPT’s capabilities, including both descriptive and inferential statistics. Data processing, categorization, and tabulation were included as assessments of data management. Each assessment was conducted in a new GPT-4 window with the memory feature disabled. For each iteration, the Microsoft Excel data file and prompt were entered sequentially into the same ChatGPT window.

### Ethical Considerations

The National Healthcare Cost and Utilization Project is a limited dataset, and it was deemed exempt by the institutional review board at the University of Arizona.

### Data Management and Descriptive Statistics

For data processing, ChatGPT was prompted to create a new variable, reclassifying “race” from 4 dimensions (White, Black, Hispanic, and Other) to 2 dimensions (White and non-White). ChatGPT was also prompted to create a subset of patients aged 45 years to facilitate nonparametric testing later on. The decision to select 45-year-olds within the original sample was arbitrary, intended solely to create a smaller subpopulation suitable for nonparametric tests. For categorization, ChatGPT was prompted to categorize patients into 3 age cohorts (41-50, 51-60, and 61-70 years). Finally, data tabulation involved creating a series table to display the frequencies for the variables used in the categorization and processing steps. The prompts used for each element of data processing and categorization can be found in Multimedia Appendix 1.

For descriptive statistics, the methods tested were mean, SD, median, and IQR. ChatGPT was prompted to compute these values for male and female cohorts, as well as the entire dataset for the following variables: age, length of stay, and total charges. All descriptive statistics prompts were posed as directed questions without opportunity for interpretation. Descriptive statistics prompts can be found in Multimedia Appendix 2.

### Inferential Statistics

Inferential statistics were performed as both parametric tests (chi-square, Pearson correlation, independent 2-sample *t* test, and 1-way ANOVA) and equivalent nonparametric tests (Fisher exact, Spearman rank order correlation, Mann-Whitney *U* test, and Kruskal-Wallis *H* test). The pairings of statistical tests are displayed in Table 1. A series of research questions were developed to guide statistical method selection (MS) toward a specific test, as shown in Table 2. Once refined, each research question was posed to ensure that a 2-tailed analysis was conducted, rather than a 1-tailed. The research questions and their proposed analytical tests were selected by one of the authors, a biostatistician, within the medical school.

**Table 1:** Parametric and nonparametric statistical analysis pairs.

Parametric test	Nonparametric test
Chi-square^a^	Fisher exact
Pearson correlation	Spearman rank order correlation
Independent 2-sample *t* test	Mann-Whitney *U*
1-way ANOVA	Kruskal-Wallis *H*

^a^Chi-square is a nonparametric test, as it does not require the assumption of normality. However, like the other tests listed in the “Parametric test” column, chi-square is better suited for larger sample sizes compared with its counterpart in the “Nonparametric test” column.

**Table 2:** Research questions and expected results.

Question	Method selection	Statistical assumptions	Statistical values
Is the distribution of White and non-White patients the same across genders?	Chi-square	Independence Expected values ≥5	*χ*^2^ (*df*) → 1.385 (1) *P*=.24
Is the distribution of White and non-White patients the same across genders for individuals who are 45 years old?	Fisher exact	Independence	Odds ratio=2.045 *P*=.66
Is there a significant correlation between total charges and length of stay?^a^	Pearson correlation	Linearity Normality Homoscedasticity No extreme outliers Paired data	*r*=0.842 *P*<.001
Is there a significant correlation between total charges and length of stay for individuals who are 45 years old?	Spearman rank order correlation	Monotonicity Nonnominal data	ρ=0.447 *P*=.02
Is there a significant difference in total charges between men and women?	Independent 2-sample *t* test	Independence Normality Homoscedasticity No extreme outliers Continuous data	*t*-stat (*df*) → 0.185 (2707) *P*=.85
Is there a significant difference in total charges between men and women who are 45 years old?	Mann-Whitney *U*	Independence	*U*-stat=91.000^b^ *P*=.17
Are there significant differences in length of stay across race categories?	1-way ANOVA	Independence Normality Homoscedasticity No extreme outliers Continuous data	*F*-stat=4.092 *P*=.007
Are there significant differences in length of stay across race categories^c^ for individuals who are 45 years old?	Kruskal-Wallis *H*	Independence	*H*-stat=1.984 *P*=.37

^a^The variables were transformed for this test to improve normality and homoscedasticity.

^b^The *U*-stat was computed in Python and RStudio only, as SAS does not produce an equivalent value.

^c^The Hispanic group was excluded from this test due to insufficient observations.

The prompts used for inferential statistics were formulated at 3 levels of increasing specificity, reflecting a user’s familiarity with statistics, ChatGPT, and Python. The lowest level was “Basic,” where ChatGPT was provided with the research question, relevant variables, and necessary tasks. Textbox 1 shows a template used for “Basic” prompts. The “Intermediate” level used prompts that were more specific than “Basic” and included additional guidelines, including data cleaning steps and strategies for assessing the statistical assumptions (SA). The “Advanced” level prompted ChatGPT with the same information as “Intermediate” and a recommended statistical test. An overview of the components for each level of prompt is provided in Table 3. The individual prompts used for each test can be found in Multimedia Appendix 3.

**A “Basic” template prompt for inferential statistics.**

I am analyzing variables related to hospital visits and demographics to answer the question, “_______?” The relevant variables are as follows:

- x (variable_type): description
- y (variable_type): description

Embody the role of an experienced biostatistician and complete the following tasks:

- Suggest the most relevant statistical method for analyzing this dataset.
- List and verify all of the critical assumptions that must be met to perform this statistical method.
- If any critical assumptions of the primary test are not met, identify a more appropriate alternative analysis. When suggesting a new test, list and verify the assumptions for this new test.
- Perform the most appropriate test and provide test statistics and *P* values to 3 decimal places.

**Table 3:** Components of inferential statistics prompts at varying knowledge levels.

	Basic	Intermediate	Advanced
Research question	✓	✓	✓
Variables and variable types	✓	✓	✓
Data clean step		✓	✓
Strategies to assess assumptions		✓	✓
Suggested method			✓
Tasks to perform (MS^a^, SA^b^, SV^c^)	✓	✓	✓

^a^MS: method selection.

^b^SA: statistical assumptions.

^c^SV: statistical values.

### Response Grading

The data analysis tasks were also performed in Python (Python Software Foundation), SAS (SAS Institute), and RStudio (Posit PBC) to generate an expected statistical output for comparison. For data processing, data categorization, data tabulation, and descriptive statistics, the expected statistical values (SV) can be found in Multimedia Appendix 4.

For inferential statistics, the expected statistical results are in the SV column of Table 2. The results were consistent among Python, SAS, and RStudio. If there were any discrepancies occurring due to algorithmic variations among the platforms, the Python output would have been designated as the “gold standard,” as ChatGPT uses this language for its calculations.

The measured outcomes were data processing, data categorization, data tabulation, descriptive statistics, and inferential statistics. A trial consisted of a single interaction with ChatGPT, including one user input (prompt) and one ChatGPT output. Data processing and categorization were assessed through a direct comparison of the frequencies generated by ChatGPT to those in Python, SAS, and RStudio, each across 10 trials. Data tabulation was assessed through ChatGPT’s ability to provide the correct columns, rows, and row totals during the processing and categorization trials (20 trials total).

Descriptive statistics were assessed through a direct comparison of the mean, SD, median, and IQR generated by ChatGPT to the expected values across 10 trials. Inferential statistics tests were evaluated on 3 criteria: MS, SA, and SV across 10 trials per statistical method (80 trials total). MS involved selecting the expected method, SA involved reporting the underlying assumptions critical to the statistical test, and SV involved calculating the test statistics and *P* values for the selected statistical method.

For data processing, data categorization, data tabulation, and descriptive statistics, the trial was coded as correct only if all values from ChatGPT’s output were within 1% of the expected values. For inferential statistics, MS was coded as correct only if the final test selected by ChatGPT matched the expected test, SA was coded as correct only if the expected assumptions were assessed implicitly or explicitly, and SV was coded as correct only if both the test statistic and *P* value were within 1% of the expected output. A flowchart detailing how all trials were assessed is available in Multimedia Appendix 5.

All measured outcomes were coded as either correct or incorrect. Only a single prompt was provided to ChatGPT in each trial, with no further interaction. If ChatGPT requested further user input or provided multiple answers without indicating the best choice, that trial was coded as incorrect. If ChatGPT timed out during code interpretation, that trial was discarded. During inferential statistics trials, if MS was incorrect, then SA and SV were coded as incorrect as well. The choice of 10 trials per outcome was informed by previous studies on ChatGPT’s performance in data analysis tasks, most of which assessed ChatGPT’s performance in fewer trials [6,7,11]. In the absence of an established standard, 10 trials were selected to capture variability in ChatGPT’s responses.

## Results

### Data Management and Descriptive Statistics

ChatGPT performed data processing, categorization, and tabulation correctly across all trials. Variations in table aesthetics were inconsequential to the measured outcomes. For descriptive statistics, ChatGPT provided accurate means, SDs, medians, and IQRs for the 3 continuous variables across all attempts. The compiled tables and descriptive statistics for each variable can be found in Multimedia Appendix 4.

### Inferential Statistics

**Figure 1:**
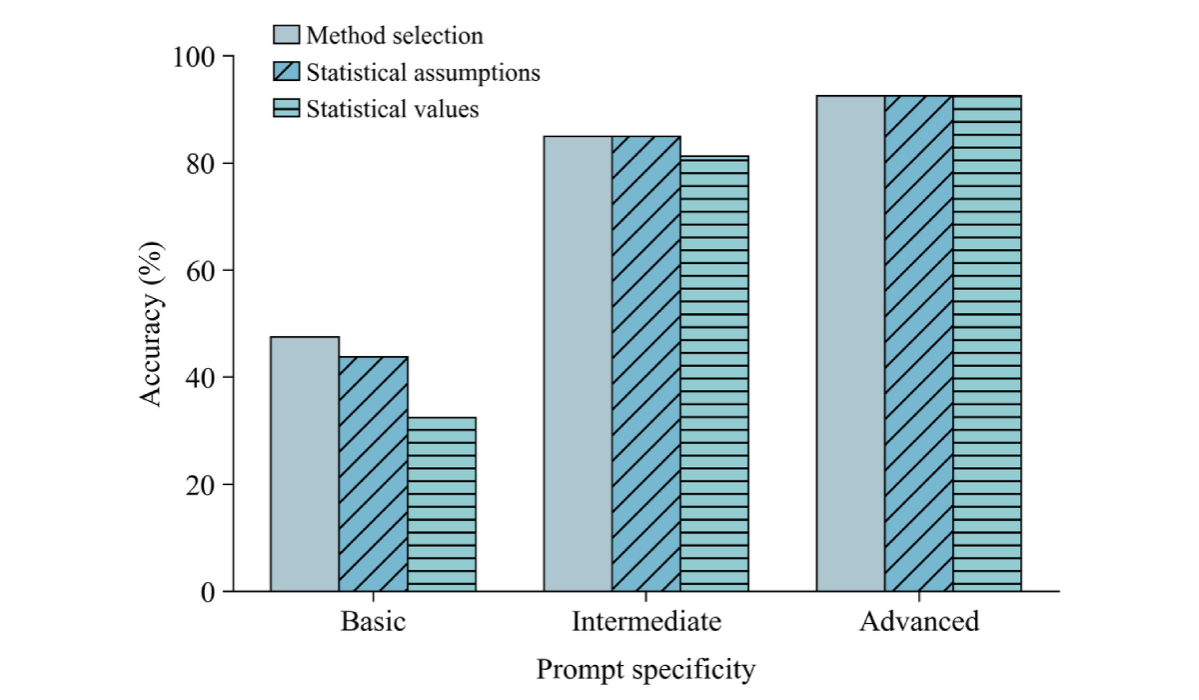
ChatGPT accuracy by prompt specificity.

When provided a “Basic” prompt, ChatGPT achieved 47.5% accuracy (38/80 attempts) on MS, 43.8% accuracy (35/80 attempts) on SA, and 32.5% accuracy (26/80 attempts) on SV. With “Intermediate” prompts, ChatGPT achieved 85.0% accuracy (68/80 attempts) on both MS and SA and 81.3% accuracy (65/80 attempts) on SV. With “Advanced” prompts, ChatGPT achieved 92.5% accuracy (74/80 attempts) across all MS, SA, and SV assessments. These results are displayed in Table 4 and Figure 1.

**Table 4:** Results for 10 attempts at inferential statistics with prompts at varying levels of specificity.

Test	Basic				Intermediate				Advanced		
	MS^a^	SA^b^	SV^c^	MS		SA	SV	MS		SA	SV
Chi-square	10	10	0	10		10	7	10		10	10
Fisher	9	9	9	10		10	10	10		10	10
Pearson	2	2	2	8		8	8	8		8	8
Spearman	9	6	8	7		7	7	10		10	10
*t* test	0	0	0	10		10	10	9		9	9
Mann-Whitney	7	7	6	8		8	8	9		9	9
ANOVA	1	1	1	7		7	7	8		8	8
Kruskal-Wallis	0	0	0	8		8	8	10		10	10

^a^MS: method selection.

^b^SA: statistical assumptions.

^c^SV: statistical values.

The errors encountered by ChatGPT across all inferential statistics trials included incorrect MS (n=51), coding error (n=13), incomplete response (n=9), incomplete assumptions (n=2), median imputed for missing value (n=1), and outliers incorrectly removed (n=1). A complete list of errors is listed in Table 5. Coding errors were limited to chi-square trials only and occurred due to a feature of the Python function. This function, chi2_contigency, contains a parameter for performing Yates correction, a method to prevent overestimation with small samples. As the parameter defaults to true, ChatGPT performed this correction despite the large sample size.

**Table 5:** Frequency of errors encountered by ChatGPT during inferential statistics trials.

Test	Basic	Intermediate	Advanced
Chi-square	Coding error: 10	Coding error: 3	—^a^
Fisher	Incorrect method: 1	—	—
Pearson	Incorrect method: 7 Incomplete response: 1	Incorrect method: 2	Incorrect method: 2
Spearman	Incomplete assumptions: 2 Incomplete response: 1 Imputed value: 1	Incorrect method: 2 Incomplete response: 1	—
*t* test	Incorrect method: 10	—	Incorrect method: 1
Mann-Whitney	Incomplete response: 3 Outliers removed: 1	Incorrect method: 2	Incomplete response: 1
ANOVA	Incorrect method: 8 Incomplete response: 1	Incorrect method: 3	Incorrect method: 2
Kruskal-Wallis	Incorrect method: 10	Incorrect method: 1 Incomplete response: 1	—

^a^Not applicable.

## Discussion

### Principal Findings

Our investigation into the analytical capabilities of ChatGPT revealed a nuanced understanding of its implementation that, while promising, exhibits limitations that warrant careful consideration. ChatGPT’s performance demonstrated proficiency in data processing, data categorization, data tabulation, and descriptive statistics. The most specific prompts improved response accuracy across inferential statistics trials. Based on our data, “Basic” prompts provided little value, with a low overall accuracy across all trials. Meanwhile, “Intermediate” prompts resulted in a similar overall accuracy compared with “Advanced” prompts despite the addition of a suggested statistical test in the “Advanced” prompts.

The variation observed across trials reflects the probabilistic nature and inherent unpredictability of ChatGPT [15]. Evaluating its accuracy as a tool requires repeated testing [16]. Given that GPT-4 is trained on over 1 trillion parameters [17], a definitive assessment of its performance is nearly impossible, as it would require countless trials to capture all possible response variations. This could explain the anomalies in the *t* test and Spearman trials, where less specific prompts sometimes outperformed more detailed ones.

The calculation errors from ChatGPT likely stem from challenges in implementing user instructions and interpreting intermediary outputs, rather than inherent issues with the Python-based analytical frameworks. With intentional instructions, most inaccuracies can be avoided. Although MS was the most common source of error, ChatGPT’s responses included sufficient information for users to verify the suitability of the statistical method. Furthermore, in the “Intermediate” and “Advanced” trials, if ChatGPT selected the correct method, the remaining tasks were frequently completed accurately. Precise and specific prompts generally enhance accuracy; however, erroneous instructions may introduce further inaccuracies, as ChatGPT heavily relies on user input. Successful use of ChatGPT as a statistical doula requires balancing clear analysis goals with the flexibility needed for ChatGPT to present accurate information, without inadvertently introducing bias.

The power and democratization of ChatGPT allow for collaboration to further mitigate concerns about the analytical process. Although these trials were based on single prompts, a series of follow-up prompts by a user can identify determinations with regard to the data, the proposed analysis, or the code. For example, when asked about the Yates correction, ChatGPT acknowledged its use for small samples. To prevent errors from defaulted values like the Yates parameter, prompts should instruct ChatGPT to specify a function’s parameters and provide the rationale for setting each parameter. This method was incorporated into the “Intermediate” and “Advanced” trials.

ChatGPT will inevitably produce inaccurate results that, if not carefully verified, may be detrimental to the research community. Therefore, all ChatGPT-generated analyses should be approached with caution. Based on this 10-trial approach, researchers are encouraged to incorporate multiple trials of the requested analysis in the statistical workflow using as specific instructions as possible. ChatGPT is not intended to be a standalone tool for data analysis; consultation with a biostatistician is essential for validating statistical approaches and ensuring reliable results.

Our dataset and assessments are limitations. Although we selected a moderately sized sample, it is still small relative to datasets used in complex analyses and may not assess the extent of ChatGPT’s capabilities. In this report, the statistical analyses focused on univariate comparisons, where the capabilities for bivariate data analysis remain to be validated. Furthermore, due to the probabilistic nature of ChatGPT, a definitive performance assessment would require thousands of trials. Finally, we did not assess the validity of ChatGPT’s statements outside of the chosen measures. When ChatGPT provided correct, incorrect, or extraneous information in the output, we did not count that against the assessment of accuracy to replicate the expected analysis.

### Conclusion

ChatGPT has significant potential as a tool for exploratory data analysis, particularly for researchers who have some statistical knowledge but limited programming expertise. This paper is intended for individuals with a foundational understanding of statistics who could generate “Intermediate” prompts on their own. We hope that these individuals may use ChatGPT to perform preliminary analyses, helping them to understand their data, draw initial insights, and begin writing their papers while waiting for consultation with a statistician. These preliminary analyses are not meant to replace expert review but to accelerate the research process. Furthermore, ChatGPT may serve as an educational resource, helping researchers better understand statistical analyses and tackle unique problems [18].

Further advancements to ChatGPT will undoubtedly enhance its applicability and accuracy in statistical analysis. Data visualization is a crucial component of data analysis, and although still limited, ChatGPT’s ability to process visual inputs through its “Vision” feature has already shown promise in interpreting statistical figures [19]. Furthermore, OpenAI is rapidly advancing its GPT models, and GPT-o1, the latest version, is already available for preview. This new model is reportedly better suited to “reason through complex tasks and solve more difficult problems in science, coding, and math” [20]. Improvements to both “Vision” and the GPT model will solidify ChatGPT’s role as an asset for researchers.

We encourage researchers to leverage ChatGPT for the programming aspects of their statistical work while leaving the critical decisions to human expertise. By doing so, they can harness the full potential of ChatGPT as a supplementary tool in their research arsenal, ensuring that its application is both productive and scientifically sound. For researchers interested in applying ChatGPT to data analysis, we recommend following the best practices outlined in Textbox 2.

**Best practices for performing data analysis with ChatGPT.**

**Prompting**

- Prompts should have specific and comprehensive goals. Researchers can benefit from creating a flexible template that can be adapted across different data analysis tasks. Additional details relevant to the current query should be included as needed. For those using ChatGPT for inferential statistics, prompts should, at a minimum, resemble the level of detail used in our “Intermediate” trials.

**Refinement**

- Approach data analysis like a discussion. Engage ChatGPT by asking it to identify any potential gaps in its knowledge or the inputs provided. Request feedback on how prompts can be improved for future trials. Ask ChatGPT to explain any decisions or necessary parameters.

**Consistency**

- At a minimum, attempt each trial 3 times to verify its consistency.

**Verification**

- ChatGPT-generated data analysis can be used to start drafting a paper before consulting a statistician. However, ensure that all statistical outputs and interpretations are reviewed by a statistician before publication.

**Transparency**

- Be transparent about using ChatGPT to assist with data analysis. Consider sharing the prompts used in your analysis to allow readers to replicate or refine the process.

## Appendix

Multimedia Appendix 1 Data processing and categorization prompts.

Multimedia Appendix 2 Descriptive statistics prompts.

Multimedia Appendix 3 Inferential statistics prompts at varying levels of specificity.

Multimedia Appendix 4 Data tabulation and descriptive statistics results.

Multimedia Appendix 5 Trial design flowchart.

## Abbreviations

AI: artificial intelligence
MS: method selection
SA: statistical assumptions
SV: statistical values
