# Supplementary material for: ChatGPT for Univariate Statistics: Validation of AI-Assisted Data Analysis in Healthcare Research
Source: J Med Internet Res. 2025 Feb 7;27:e63550. doi: 10.2196/63550 (PMC11845875; doi:10.2196/63550)
Supplement: Multimedia Appendix 1 [file jmir_v27i1e63550_app1.docx]

**DATA PROCESSING**

I am analyzing the following variables related to demographics and gender from a dataset:

- RACE (categorical): “1” = white; “2” = black; “3” = hispanic; “4” = other

- RACE_BINARY (categorical): “1” = white; “2” = non-white

- FEMALE (categorical): “0” = male; “1” = female

- AGE (continuous): numerical age

Complete the following tasks:

1. Create a table showing the frequency of each race_cat category broken down by gender (male, female), age (specifically 45-year-olds), and for the entire dataset.

- The table should include both the count and proportion for each category within these groups.

- Use the following headers for rows: "All", "Men", "Women", and "45-year-olds"; and for columns: "White", "Black", "Hispanic", "Other", and "Total" (in this order; descending and left-to-right, respectively).

2. Similarly, create a table for the race_bin variable with the same breakdowns as above.

- The columns should be "White", "Non-White", and "Total" (in this order).

Expected Output Format: Each entry in the tables should contain the frequency followed by the proportion in parentheses. Do not include proportions in the "Total" column, only the frequency. Proportions should be expressed as percentages rounded to one decimal place.

**DATA CATEGORIZATION**

I am analyzing the following variables related to demographics and gender from a dataset:

- FEMALE (categorical): “0” = male; “1” = female

- AGE (continuous): numerical age

Complete the following tasks:

1. Subset the age variable into three cohorts: 41-50, 51-60, and 61-70.

2. Create a table showing the frequency of each age cohort broken down by gender (male, female) and for the entire dataset.

- The table should include both the count and proportion for each category within these groups.

- Use the following headers for rows: "All", "Men", and "Women"; and for columns: "41-50", "51-60", and "61-70", and "Total" (in this order; descending and left-to-right, respectively).

Expected Output Format: Each entry in the tables should contain the frequency followed by the proportion in parentheses. Do not include proportions in the "Total" column, only the frequency. Proportions should be expressed as percentages rounded to one decimal place.
