# Supplementary material for: ChatGPT for Univariate Statistics: Validation of AI-Assisted Data Analysis in Healthcare Research
Source: J Med Internet Res. 2025 Feb 7;27:e63550. doi: 10.2196/63550 (PMC11845875; doi:10.2196/63550)
Supplement: Multimedia Appendix 2 [file jmir_v27i1e63550_app2.docx]

**DESCRIPTIVE STATISTICS**

I am analyzing the following variables related to hospital visits and demographics from a dataset:

- LOS (continuous): a patient’s length of stay in number of days

- TOTCHG (continuous): a patient’s dollar amount of total billed charges

- FEMALE (categorical): “0” = male; “1” = female

- AGE (continuous): numerical age

Complete the following tasks:

1. For patient age, provide the mean, standard deviation, median, and interquartile range broken down by category for men, women, and the entire dataset.

2. Repeat step 1. for length of stay and total charges.

Expected Output Format: Provide all values to two decimal places.
