# Supplementary material for: ChatGPT for Univariate Statistics: Validation of AI-Assisted Data Analysis in Healthcare Research
Source: J Med Internet Res. 2025 Feb 7;27:e63550. doi: 10.2196/63550 (PMC11845875; doi:10.2196/63550)
Supplement: Multimedia Appendix 3 [file jmir_v27i1e63550_app3.docx]

**CHI SQUARE**

**Basic:**

I am analyzing variables related to hospital visits and demographics to answer the question, “Is the distribution of White and Non-White patients the same across genders?” The relevant variables are as follows:

- FEMALE (categorical): “0” = male; “1” = female
- RACE_BINARY (categorical): “1” = white; “2” = non-white

Embody the role of an experienced biostatistician and complete the following tasks:

1. Suggest the most relevant statistical method to use for analyzing this dataset.

2. List and verify all of the critical assumptions that must be met in order to perform this statistical method.

3. If any critical assumptions of the primary test are not met, identify a more appropriate alternative analysis. If suggesting a new test, list and verify the assumptions for this new test.

4. Perform the most appropriate test and provide test statistics and p-values to 3 decimal places.

**Intermediate:**

I am analyzing variables related to hospital visits and demographics to answer the question, “Is the distribution of White and Non-White patients the same across genders?” The relevant variables are as follows:

- FEMALE (categorical): “0” = male; “1” = female
- RACE_BINARY (categorical): “1” = white; “2” = non-white

Embody the role of an experienced biostatistician and complete the following tasks:

1. Load the data, filtering only for relevant entries. Perform a data clean by removing NaN/blank entries in the relevant variables. Calculate the sample size for the cleaned and filtered dataset and store this value as a variable named sample_size.

2. Suggest the most relevant statistical method to use for analyzing this dataset, considering both the research question, nature of the data, and sample size.

3. Rigorously list all of the critical assumptions that must be met in order to perform this statistical method.

4. Load the necessary libraries to perform the statistical functions and begin assessing these assumptions within the context of the dataset. Show all tables/figures/calculations necessary to assess the assumptions. Keep the following in mind:

- Do not perform any additional transformations on the data.
- If the test involves a minimum expected value, store the expected values in an array named exp_value. Perform an iterative function that goes through each entry in the exp_value array to ensure that the expected value criteria is met.

5. If any critical assumptions of the primary test are not met, identify a more appropriate alternative analysis. While some flexibility is allowed with less critical assumptions, ensure that the selected test provides valid and reliable results given the data characteristics. List the assumptions that are necessary for this alternative test, and based on your calculations determine if these have been met as well.

6. List the assumptions for the most appropriate test (list them again if the most appropriate test was the first one selected). Carefully examine all calculated values and re-verify that each of the assumptions is met. Provide explicit justifications for each assumption with any numeric values listed clearly.

7. Select only the most appropriate test based on the thoroughly evaluated assumptions. For this test, list all of the parameters of the python function. Analyze these parameters and select the appropriate inputs that match the nature of our dataset and question. Ensure that none of the parameters have been automatically set to be true when they are not intended to be. Perform this selected test and provide test statistics and p-values to 3 decimal places. Do not stop until the test is complete.

**Advanced:**

I am analyzing variables related to hospital visits and demographics to answer the question, “Is the distribution of White and Non-White patients the same across genders?” The relevant variables are as follows:

- FEMALE (categorical): “0” = male; “1” = female
- RACE_BINARY (categorical): “1” = white; “2” = non-white

Embody the role of an experienced biostatistician and complete the following tasks:

1. Load the data, filtering only for relevant entries. Perform a data clean by removing NaN/blank entries in the relevant variables. Calculate the sample size for the cleaned and filtered dataset and store this value as a variable named sample_size.

2. Suggest the most relevant statistical method to use for analyzing this dataset, considering both the research question, nature of the data, and sample size. I believe the best choice would be Chi square.

3. Rigorously list all of the critical assumptions that must be met in order to perform this statistical method.

4. Load the necessary libraries to perform the statistical functions and begin assessing these assumptions within the context of the dataset. Show all tables/figures/calculations necessary to assess the assumptions. Keep the following in mind:

- Do not perform any additional transformations on the data.

- If the test involves a minimum expected value, store the expected values in an array named exp_value. Perform an iterative function that goes through each entry in the exp_value array to ensure that the expected value criteria is met.

5. If any critical assumptions of the primary test are not met, identify a more appropriate alternative analysis. While some flexibility is allowed with less critical assumptions, ensure that the selected test provides valid and reliable results given the data characteristics. List the assumptions that are necessary for this alternative test, and based on your calculations determine if these have been met as well.

6. List the assumptions for the most appropriate test (list them again if the most appropriate test was the first one selected). Carefully examine all calculated values and re-verify that each of the assumptions is met. Provide explicit justifications for each assumption with any numeric values listed clearly.

7. Select only the most appropriate test based on the thoroughly evaluated assumptions. For this test, list all of the parameters of the python function. Analyze these parameters and select the appropriate inputs that match the nature of our dataset and question. Ensure that none of the parameters have been automatically set to be true when they are not intended to be. If you agree with my choice of Chi Square, perform this test (ensure the Yates parameter is set to False) and provide test statistics and p-values to 3 decimal places. Do not stop until the test is complete.

**FISHER’S EXACT**

**Basic:**

I am analyzing variables related to hospital visits and demographics to answer the question, “Is the distribution of White and Non-White patients the same across genders for individuals who are 45 years old?” The relevant variables are as follows:

- FEMALE (categorical): “0” = male; “1” = female
- RACE_BINARY (categorical): “1” = white; “2” = non-white
- AGE (continuous): numerical age

Embody the role of an experienced biostatistician and complete the following tasks:

1. Suggest the most relevant statistical method to use for analyzing this dataset.

2. List and verify all of the critical assumptions that must be met in order to perform this statistical method.

3. If any critical assumptions of the primary test are not met, identify a more appropriate alternative analysis. If suggesting a new test, list and verify the assumptions for this new test.

4. Perform the most appropriate test and provide test statistics and p-values to 3 decimal places.

**Intermediate:**

I am analyzing variables related to hospital visits and demographics to answer the question, “Is the distribution of White and Non-White patients the same across genders for individuals who are 45 years old?” The relevant variables are as follows:

- FEMALE (categorical): “0” = male; “1” = female
- RACE_BINARY (categorical): “1” = white; “2” = non-white
- AGE (continuous): numerical age

Embody the role of an experienced biostatistician and complete the following tasks:

1. Load the data, filtering only for relevant entries. Perform a data clean by removing NaN/blank entries in the relevant variables. Calculate the sample size for the cleaned and filtered dataset and store this value as a variable named sample_size.

2. Suggest the most relevant statistical method to use for analyzing this dataset, considering both the research question, nature of the data, and sample size.

3. Rigorously list all of the critical assumptions that must be met in order to perform this statistical method.

4. Load the necessary libraries to perform the statistical functions and begin assessing these assumptions within the context of the dataset. Show all tables/figures/calculations necessary to assess the assumptions. Keep the following in mind:

- Do not perform any additional transformations on the data.

- If the test involves a minimum expected value, store the expected values in an array named exp_value. Perform an iterative function that goes through each entry in the exp_value array to ensure that the expected value criteria is met.

5. If any critical assumptions of the primary test are not met, identify a more appropriate alternative analysis. While some flexibility is allowed with less critical assumptions, ensure that the selected test provides valid and reliable results given the data characteristics. List the assumptions that are necessary for this alternative test, and based on your calculations determine if these have been met as well.

6. List the assumptions for the most appropriate test (list them again if the most appropriate test was the first one selected). Carefully examine all calculated values and re-verify that each of the assumptions is met. Provide explicit justifications for each assumption with any numeric values listed clearly.

7. Select only the most appropriate test based on the thoroughly evaluated assumptions. For this test, list all of the parameters of the python function. Analyze these parameters and select the appropriate inputs that match the nature of our dataset and question. Ensure that none of the parameters have been automatically set to be true when they are not intended to be. Perform this selected test and provide test statistics and p-values to 3 decimal places. Do not stop until the test is complete.

**Advanced:**

I am analyzing variables related to hospital visits and demographics to answer the question, “Is the distribution of White and Non-White patients the same across genders for individuals who are 45 years old?” The relevant variables are as follows:

- FEMALE (categorical): “0” = male; “1” = female
- RACE_BINARY (categorical): “1” = white; “2” = non-white
- AGE (continuous): numerical age

Embody the role of an experienced biostatistician and complete the following tasks:

1. Load the data, filtering only for relevant entries. Perform a data clean by removing NaN/blank entries in the relevant variables. Calculate the sample size for the cleaned and filtered dataset and store this value as a variable named sample_size.

2. Suggest the most relevant statistical method to use for analyzing this dataset, considering both the research question, nature of the data, and sample size. I believe the best choice would be Fisher’s exact.

3. Rigorously list all of the critical assumptions that must be met in order to perform this statistical method.

4. Load the necessary libraries to perform the statistical functions and begin assessing these assumptions within the context of the dataset. Show all tables/figures/calculations necessary to assess the assumptions. Keep the following in mind:

- Do not perform any additional transformations on the data.

- If the test involves a minimum expected value, store the expected values in an array named exp_value. Perform an iterative function that goes through each entry in the exp_value array to ensure that the expected value criteria is met.

5. If any critical assumptions of the primary test are not met, identify a more appropriate alternative analysis. While some flexibility is allowed with less critical assumptions, ensure that the selected test provides valid and reliable results given the data characteristics. List the assumptions that are necessary for this alternative test, and based on your calculations determine if these have been met as well.

6. List the assumptions for the most appropriate test (list them again if the most appropriate test was the first one selected). Carefully examine all calculated values and re-verify that each of the assumptions is met. Provide explicit justifications for each assumption with any numeric values listed clearly.

7. Select only the most appropriate test based on the thoroughly evaluated assumptions. For this test, list all of the parameters of the python function. Analyze these parameters and select the appropriate inputs that match the nature of our dataset and question. Ensure that none of the parameters have been automatically set to be true when they are not intended to be. If you agree with my choice of Fisher’s exact, perform this test and provide test statistics and p-values to 3 decimal places. Do not stop until the test is complete.

**PEARSON CORRELATION**

**Basic:**

I am analyzing variables related to hospital visits and demographics to answer the question, “Is there a significant correlation between total charges and length of stay?” The relevant variables are as follows:

- tra_LOS (continuous): a patient’s length of stay in number of days

- tra_TOTCHG (continuous): a patient’s dollar amount of total billed charges

Embody the role of an experienced biostatistician and complete the following tasks:

1. Suggest the most relevant statistical method to use for analyzing this dataset.

2. List and verify all of the critical assumptions that must be met in order to perform this statistical method.

3. If any critical assumptions of the primary test are not met, identify a more appropriate alternative analysis. If suggesting a new test, list and verify the assumptions for this new test.

4. Perform the most appropriate test and provide test statistics and p-values to 3 decimal places.

**Intermediate:**I am analyzing variables related to hospital visits and demographics to answer the question, “Is there a significant correlation between total charges and length of stay?” The relevant variables are as follows:

- tra_LOS (continuous): a patient’s length of stay in number of days

- tra_TOTCHG (continuous): a patient’s dollar amount of total billed charges

Embody the role of an experienced biostatistician and complete the following tasks:

1. Load the data, filtering only for relevant entries. Perform a data clean by removing NaN/blank entries in the relevant variables. Calculate the sample size for the cleaned and filtered dataset and store this value as a variable named sample_size.

2. Suggest the most relevant statistical method to use for analyzing this dataset, considering both the research question, nature of the data, and sample size.

3. Rigorously list all of the critical assumptions that must be met in order to perform this statistical method.

4. Load the necessary libraries to perform the statistical functions and begin assessing these assumptions within the context of the dataset. Show all tables/figures/calculations necessary to assess the assumptions. Keep the following in mind:

- Do not perform any additional transformations on the data.

- If the test requires normality, assess this using histograms, Q-Q plots, and kurtosis/skewness calculations. Do not use the Shapiro-Wilk test.

- If the test requires homoscedasticity, assess this using the Breusch-Pagan test (ensuring you are passing the residuals model into the function).

5. If any critical assumptions of the primary test are not met, identify a more appropriate alternative analysis. While some flexibility is allowed with less critical assumptions, ensure that the selected test provides valid and reliable results given the data characteristics. List the assumptions that are necessary for this alternative test, and based on your calculations determine if these have been met as well.

6. List the assumptions for the most appropriate test (list them again if the most appropriate test was the first one selected). Carefully examine all calculated values and re-verify that each of the assumptions is met. Provide explicit justifications for each assumption with any numeric values listed clearly.

7. Select only the most appropriate test based on the thoroughly evaluated assumptions. For this test, list all of the parameters of the python function. Analyze these parameters and select the appropriate inputs that match the nature of our dataset and question. Ensure that none of the parameters have been automatically set to be true when they are not intended to be. Perform this selected test and provide test statistics and p-values to 3 decimal places. Do not stop until the test is complete.

**Advanced:**

I am analyzing variables related to hospital visits and demographics to answer the question, “Is there a significant correlation between total charges and length of stay?” The relevant variables are as follows:

- tra_LOS (continuous): a patient’s length of stay in number of days

- tra_TOTCHG (continuous): a patient’s dollar amount of total billed charges

Embody the role of an experienced biostatistician and complete the following tasks:

1. Load the data, filtering only for relevant entries. Perform a data clean by removing NaN/blank entries in the relevant variables. Calculate the sample size for the cleaned and filtered dataset and store this value as a variable named sample_size.

2. Suggest the most relevant statistical method to use for analyzing this dataset, considering both the research question, nature of the data, and sample size. I believe the best choice would be Pearson correlation.

3. Rigorously list all of the critical assumptions that must be met in order to perform this statistical method.

4. Load the necessary libraries to perform the statistical functions and begin assessing these assumptions within the context of the dataset. Show all tables/figures/calculations necessary to assess the assumptions. Keep the following in mind:

- Do not perform any additional transformations on the data.

- If the test requires normality, assess this using histograms, Q-Q plots, and kurtosis/skewness calculations. Do not use the Shapiro-Wilk test.

- If the test requires homoscedasticity, assess this using the Breusch-Pagan test (ensuring you are passing the residuals model into the function).

5. If any critical assumptions of the primary test are not met, identify a more appropriate alternative analysis. While some flexibility is allowed with less critical assumptions, ensure that the selected test provides valid and reliable results given the data characteristics. List the assumptions that are necessary for this alternative test, and based on your calculations determine if these have been met as well.

6. List the assumptions for the most appropriate test (list them again if the most appropriate test was the first one selected). Carefully examine all calculated values and re-verify that each of the assumptions is met. Provide explicit justifications for each assumption with any numeric values listed clearly.

7. Select only the most appropriate test based on the thoroughly evaluated assumptions. For this test, list all of the parameters of the python function. Analyze these parameters and select the appropriate inputs that match the nature of our dataset and question. Ensure that none of the parameters have been automatically set to be true when they are not intended to be. If you agree with my choice of Pearson correlation, perform this test and provide test statistics and p-values to 3 decimal places. Do not stop until the test is complete.

**SPEARMAN CORRELATION**

**Basic:**

I am analyzing variables related to hospital visits and demographics to answer the question, “Is there a significant correlation between total charges and length of stay for individuals who are 45 years old?” The relevant variables are as follows:

- LOS (continuous): a patient’s length of stay in number of days
- TOTCHG (continuous): a patient’s dollar amount of total billed charges
- AGE (continuous): a patient’s numerical age

Embody the role of an experienced biostatistician and complete the following tasks:

1. Suggest the most relevant statistical method to use for analyzing this dataset.

2. List and verify all of the critical assumptions that must be met in order to perform this statistical method.

3. If any critical assumptions of the primary test are not met, identify a more appropriate alternative analysis. If suggesting a new test, list and verify the assumptions for this new test.

4. Perform the most appropriate test and provide test statistics and p-values to 3 decimal places.

**Intermediate:**I am analyzing variables related to hospital visits and demographics to answer the question, “Is there a significant correlation between total charges and length of stay for individuals who are 45 years old?” The relevant variables are as follows:

- LOS (continuous): a patient’s length of stay in number of days
- TOTCHG (continuous): a patient’s dollar amount of total billed charges
- AGE (continuous): a patient’s numerical age

Embody the role of an experienced biostatistician and complete the following tasks:

1. Load the data, filtering only for relevant entries. Perform a data clean by removing NaN/blank entries in the relevant variables. Calculate the sample size for the cleaned and filtered dataset and store this value as a variable named sample_size.

2. Suggest the most relevant statistical method to use for analyzing this dataset, considering both the research question, nature of the data, and sample size.

3. Rigorously list all of the critical assumptions that must be met in order to perform this statistical method.

4. Load the necessary libraries to perform the statistical functions and begin assessing these assumptions within the context of the dataset. Show all tables/figures/calculations necessary to assess the assumptions. Keep the following in mind:

- Do not perform any additional transformations on the data.

- If the test requires normality, assess this using histograms, Q-Q plots, and kurtosis/skewness calculations. Do not use the Shapiro-Wilk test.

- If the test requires homoscedasticity, assess this using the Breusch-Pagan test (ensuring you are passing the residuals model into the function).

5. If any critical assumptions of the primary test are not met, identify a more appropriate alternative analysis. While some flexibility is allowed with less critical assumptions, ensure that the selected test provides valid and reliable results given the data characteristics. List the assumptions that are necessary for this alternative test, and based on your calculations determine if these have been met as well.

6. List the assumptions for the most appropriate test (list them again if the most appropriate test was the first one selected). Carefully examine all calculated values and re-verify that each of the assumptions is met. Provide explicit justifications for each assumption with any numeric values listed clearly.

7. Select only the most appropriate test based on the thoroughly evaluated assumptions. For this test, list all of the parameters of the python function. Analyze these parameters and select the appropriate inputs that match the nature of our dataset and question. Ensure that none of the parameters have been automatically set to be true when they are not intended to be. Perform this selected test and provide test statistics and p-values to 3 decimal places. Do not stop until the test is complete.

**Advanced:**

I am analyzing variables related to hospital visits and demographics to answer the question, “Is there a significant correlation between total charges and length of stay for individuals who are 45 years old?” The relevant variables are as follows:

- LOS (continuous): a patient’s length of stay in number of days
- TOTCHG (continuous): a patient’s dollar amount of total billed charges
- AGE (continuous): a patient’s numerical age

Embody the role of an experienced biostatistician and complete the following tasks:

1. Load the data, filtering only for relevant entries. Perform a data clean by removing NaN/blank entries in the relevant variables. Calculate the sample size for the cleaned and filtered dataset and store this value as a variable named sample_size.

2. Suggest the most relevant statistical method to use for analyzing this dataset, considering both the research question, nature of the data, and sample size. I believe the best choice would be Spearman correlation.

3. Rigorously list all of the critical assumptions that must be met in order to perform this statistical method.

4. Load the necessary libraries to perform the statistical functions and begin assessing these assumptions within the context of the dataset. Show all tables/figures/calculations necessary to assess the assumptions. Keep the following in mind:

- Do not perform any additional transformations on the data.

- If the test requires normality, assess this using histograms, Q-Q plots, and kurtosis/skewness calculations. Do not use the Shapiro-Wilk test.

- If the test requires homoscedasticity, assess this using the Breusch-Pagan test (ensuring you are passing the residuals model into the function).

5. If any critical assumptions of the primary test are not met, identify a more appropriate alternative analysis. While some flexibility is allowed with less critical assumptions, ensure that the selected test provides valid and reliable results given the data characteristics. List the assumptions that are necessary for this alternative test, and based on your calculations determine if these have been met as well.

6. List the assumptions for the most appropriate test (list them again if the most appropriate test was the first one selected). Carefully examine all calculated values and re-verify that each of the assumptions is met. Provide explicit justifications for each assumption with any numeric values listed clearly.

7. Select only the most appropriate test based on the thoroughly evaluated assumptions. For this test, list all of the parameters of the python function. Analyze these parameters and select the appropriate inputs that match the nature of our dataset and question. Ensure that none of the parameters have been automatically set to be true when they are not intended to be. If you agree with my choice of Spearman correlation, perform this test and provide test statistics and p-values to 3 decimal places. Do not stop until the test is complete.

**INDEPENDENT TWO SAMPLE T-TEST**

**Basic:**

I am analyzing variables related to hospital visits and demographics to answer the question, “Is there a significant difference in total charges between men and women?” The relevant variables are as follows:

- TOTCHG (continuous): a patient’s dollar amount of total billed charges
- FEMALE (categorical): “0” = male; “1” = female

Embody the role of an experienced biostatistician and complete the following tasks:

1. Suggest the most relevant statistical method to use for analyzing this dataset.

2. List and verify all of the critical assumptions that must be met in order to perform this statistical method.

3. If any critical assumptions of the primary test are not met, identify a more appropriate alternative analysis. If suggesting a new test, list and verify the assumptions for this new test.

4. Perform the most appropriate test and provide test statistics and p-values to 3 decimal places.

**Intermediate:**

I am analyzing variables related to hospital visits and demographics to answer the question, “Is there a significant difference in total charges between men and women?” The relevant variables are as follows:

- TOTCHG (continuous): a patient’s dollar amount of total billed charges
- FEMALE (categorical): “0” = male; “1” = female

Embody the role of an experienced biostatistician and complete the following tasks:

1. Load the data, filtering only for relevant entries. Perform a data clean by removing NaN/blank entries in the relevant variables. Calculate the sample size for the cleaned and filtered dataset and store this value as a variable named sample_size. Calculate the size of each group, and store these as variables named {group}_size, where {group} is the name of each individual group.

2. Suggest the most relevant statistical method to use for analyzing this dataset, considering both the research question, nature of the data, and sample size.

3. Rigorously list all of the critical assumptions that must be met in order to perform this statistical method.

4. Load the necessary libraries to perform the statistical functions and begin assessing these assumptions within the context of the dataset. Show all tables/figures/calculations necessary to assess the assumptions. Keep the following in mind:

- Do not perform any additional transformations on the data.

- If the test requires normality, assess this using histograms and Q-Q plots. Do not use the Shapiro-Wilk test. However, if the Central Limit Theorem is satisfied for each individual group (and not just the entire sample), no further testing for normality is required. Use {group}_size to assess this.

- If the test requires homoscedasticity, assess this using Levene’s test.

5. If any critical assumptions of the primary test are not met, identify a more appropriate alternative analysis. While some flexibility is allowed with less critical assumptions, ensure that the selected test provides valid and reliable results given the data characteristics. List the assumptions that are necessary for this alternative test, and based on your calculations determine if these have been met as well.

6. List the assumptions for the most appropriate test (list them again if the most appropriate test was the first one selected). Carefully examine all calculated values and re-verify that each of the assumptions is met. Provide explicit justifications for each assumption with any numeric values listed clearly.

7. Select only the most appropriate test based on the thoroughly evaluated assumptions. For this test, list all of the parameters of the python function. Analyze these parameters and select the appropriate inputs that match the nature of our dataset and question. Ensure that none of the parameters have been automatically set to be true when they are not intended to be. Perform this selected test and provide test statistics and p-values to 3 decimal places. Do not stop until the test is complete.

**Advanced:**

I am analyzing variables related to hospital visits and demographics to answer the question, “Is there a significant difference in total charges between men and women?” The relevant variables are as follows:

- TOTCHG (continuous): a patient’s dollar amount of total billed charges
- FEMALE (categorical): “0” = male; “1” = female

Embody the role of an experienced biostatistician and complete the following tasks:

1. Load the data, filtering only for relevant entries. Perform a data clean by removing NaN/blank entries in the relevant variables. Calculate the sample size for the cleaned and filtered dataset and store this value as a variable named sample_size. Calculate the size of each group, and store these as variables named {group}_size, where {group} is the name of each individual group.

2. Suggest the most relevant statistical method to use for analyzing this dataset, considering both the research question, nature of the data, and sample size. I believe the best choice would be two sample t-test.

3. Rigorously list all of the critical assumptions that must be met in order to perform this statistical method.

4. Load the necessary libraries to perform the statistical functions and begin assessing these assumptions within the context of the dataset. Show all tables/figures/calculations necessary to assess the assumptions. Keep the following in mind:

- Do not perform any additional transformations on the data.

- If the test requires normality, assess this using histograms and Q-Q plots. Do not use the Shapiro-Wilk test. However, if the Central Limit Theorem is satisfied for each individual group (and not just the entire sample), no further testing for normality is required. Use {group}_size to assess this.

- If the test requires homoscedasticity, assess this using Levene’s test.

5. If any critical assumptions of the primary test are not met, identify a more appropriate alternative analysis. While some flexibility is allowed with less critical assumptions, ensure that the selected test provides valid and reliable results given the data characteristics. List the assumptions that are necessary for this alternative test, and based on your calculations determine if these have been met as well.

6. List the assumptions for the most appropriate test (list them again if the most appropriate test was the first one selected). Carefully examine all calculated values and re-verify that each of the assumptions is met. Provide explicit justifications for each assumption with any numeric values listed clearly.

7. Select only the most appropriate test based on the thoroughly evaluated assumptions. For this test, list all of the parameters of the python function. Analyze these parameters and select the appropriate inputs that match the nature of our dataset and question. Ensure that none of the parameters have been automatically set to be true when they are not intended to be. If you agree with my choice of two sample t-test, perform this test and provide test statistics and p-values to 3 decimal places. Do not stop until the test is complete.

**MANN-WHITNEY U**

**Basic:**

I am analyzing variables related to hospital visits and demographics to answer the question, “Is there a significant difference in total charges between men and women who are 45 years old?” The relevant variables are as follows:

- TOTCHG (continuous): a patient’s dollar amount of total billed charges
- FEMALE (categorical): “0” = male; “1” = female
- AGE (continuous): numerical age

Embody the role of an experienced biostatistician and complete the following tasks:

1. Suggest the most relevant statistical method to use for analyzing this dataset.

2. List and verify all of the critical assumptions that must be met in order to perform this statistical method.

3. If any critical assumptions of the primary test are not met, identify a more appropriate alternative analysis. If suggesting a new test, list and verify the assumptions for this new test.

4. Perform the most appropriate test and provide test statistics and p-values to 3 decimal places.

**Intermediate:**

I am analyzing variables related to hospital visits and demographics to answer the question, “Is there a significant difference in total charges between men and women who are 45 years old?” The relevant variables are as follows:

- TOTCHG (continuous): a patient’s dollar amount of total billed charges
- FEMALE (categorical): “0” = male; “1” = female
- AGE (continuous): numerical age

Embody the role of an experienced biostatistician and complete the following tasks:

1. Load the data, filtering only for relevant entries. Perform a data clean by removing NaN/blank entries in the relevant variables. Calculate the sample size for the cleaned and filtered dataset and store this value as a variable named sample_size. Calculate the size of each group, and store these as variables named {group}_size, where {group} is the name of each individual group.

2. Suggest the most relevant statistical method to use for analyzing this dataset, considering both the research question, nature of the data, and sample size.

3. Rigorously list all of the critical assumptions that must be met in order to perform this statistical method.

4. Load the necessary libraries to perform the statistical functions and begin assessing these assumptions within the context of the dataset. Show all tables/figures/calculations necessary to assess the assumptions. Keep the following in mind:

- Do not perform any additional transformations on the data.

- If the test requires normality, assess this using histograms and Q-Q plots. Do not use the Shapiro-Wilk test. However, if the Central Limit Theorem is satisfied for each individual group (and not just the entire sample), no further testing for normality is required. Use {group}_size to assess this.

- If the test requires homoscedasticity, assess this using Levene’s test.

5. If any critical assumptions of the primary test are not met, identify a more appropriate alternative analysis. While some flexibility is allowed with less critical assumptions, ensure that the selected test provides valid and reliable results given the data characteristics. List the assumptions that are necessary for this alternative test, and based on your calculations determine if these have been met as well.

6. List the assumptions for the most appropriate test (list them again if the most appropriate test was the first one selected). Carefully examine all calculated values and re-verify that each of the assumptions is met. Provide explicit justifications for each assumption with any numeric values listed clearly.

7. Select only the most appropriate test based on the thoroughly evaluated assumptions. For this test, list all of the parameters of the python function. Analyze these parameters and select the appropriate inputs that match the nature of our dataset and question. Ensure that none of the parameters have been automatically set to be true when they are not intended to be. Perform this selected test and provide test statistics and p-values to 3 decimal places. Do not stop until the test is complete.

**Advanced:**

I am analyzing variables related to hospital visits and demographics to answer the question, “Is there a significant difference in total charges between men and women who are 45 years old?” The relevant variables are as follows:

- TOTCHG (continuous): a patient’s dollar amount of total billed charges
- FEMALE (categorical): “0” = male; “1” = female
- AGE (continuous): numerical age

Embody the role of an experienced biostatistician and complete the following tasks:

1. Load the data, filtering only for relevant entries. Perform a data clean by removing NaN/blank entries in the relevant variables. Calculate the sample size for the cleaned and filtered dataset and store this value as a variable named sample_size. Calculate the size of each group, and store these as variables named {group}_size, where {group} is the name of each individual group.

2. Suggest the most relevant statistical method to use for analyzing this dataset, considering both the research question, nature of the data, and sample size. I believe the best choice would be Mann-Whitney U.

3. Rigorously list all of the critical assumptions that must be met in order to perform this statistical method.

4. Load the necessary libraries to perform the statistical functions and begin assessing these assumptions within the context of the dataset. Show all tables/figures/calculations necessary to assess the assumptions. Keep the following in mind:

- Do not perform any additional transformations on the data.

- If the test requires normality, assess this using histograms and Q-Q plots. Do not use the Shapiro-Wilk test. However, if the Central Limit Theorem is satisfied for each individual group (and not just the entire sample), no further testing for normality is required. Use {group}_size to assess this.

- If the test requires homoscedasticity, assess this using Levene’s test.

5. If any critical assumptions of the primary test are not met, identify a more appropriate alternative analysis. While some flexibility is allowed with less critical assumptions, ensure that the selected test provides valid and reliable results given the data characteristics. List the assumptions that are necessary for this alternative test, and based on your calculations determine if these have been met as well.

6. List the assumptions for the most appropriate test (list them again if the most appropriate test was the first one selected). Carefully examine all calculated values and re-verify that each of the assumptions is met. Provide explicit justifications for each assumption with any numeric values listed clearly.

7. Select only the most appropriate test based on the thoroughly evaluated assumptions. For this test, list all of the parameters of the python function. Analyze these parameters and select the appropriate inputs that match the nature of our dataset and question. Ensure that none of the parameters have been automatically set to be true when they are not intended to be. If you agree with my choice of Mann-Whitney U, perform this test and provide test statistics and p-values to 3 decimal places. Do not stop until the test is complete.

**ONE-WAY ANOVA**

**Basic:**

I am analyzing variables related to hospital visits and demographics to answer the question, “Are there significant differences in length of stay across race categories?” The relevant variables are as follows:

- LOS (continuous): a patient’s length of stay in number of days
- RACE (categorical): “1” = white; “2” = black; “3” = hispanic; “4” = other

Embody the role of an experienced biostatistician and complete the following tasks:

1. Suggest the most relevant statistical method to use for analyzing this dataset.

2. List and verify all of the critical assumptions that must be met in order to perform this statistical method.

3. If any critical assumptions of the primary test are not met, identify a more appropriate alternative analysis. If suggesting a new test, list and verify the assumptions for this new test.

4. Perform the most appropriate test and provide test statistics and p-values to 3 decimal places.

**Intermediate:**I am analyzing variables related to hospital visits and demographics to answer the question, “Are there significant differences in length of stay across race categories?” The relevant variables are as follows:

- LOS (continuous): a patient’s length of stay in number of days
- RACE (categorical): “1” = white; “2” = black; “3” = hispanic; “4” = other

Embody the role of an experienced biostatistician and complete the following tasks:

1. Load the data, filtering only for relevant entries. Perform a data clean by removing NaN/blank entries in the relevant variables. Calculate the sample size for the cleaned and filtered dataset and store this value as a variable named sample_size. Calculate the size of each group, and store these as variables named {group}_size, where {group} is the name of each individual group.

2. Suggest the most relevant statistical method to use for analyzing this dataset, considering both the research question, nature of the data, and sample size.

3. Rigorously list all of the critical assumptions that must be met in order to perform this statistical method.

4. Load the necessary libraries to perform the statistical functions and begin assessing these assumptions within the context of the dataset. Show all tables/figures/calculations necessary to assess the assumptions. Keep the following in mind:

- Do not perform any additional transformations on the data.

- If the test requires normality, assess this using histograms and Q-Q plots. Do not use the Shapiro-Wilk test. However, if the Central Limit Theorem is satisfied for each individual group (and not just the entire sample), no further testing for normality is required. Use {group}_size to assess this.

- If the test requires homoscedasticity, assess this using Levene’s test.

5. If any critical assumptions of the primary test are not met, identify a more appropriate alternative analysis. While some flexibility is allowed with less critical assumptions, ensure that the selected test provides valid and reliable results given the data characteristics. List the assumptions that are necessary for this alternative test, and based on your calculations determine if these have been met as well.

6. List the assumptions for the most appropriate test (list them again if the most appropriate test was the first one selected). Carefully examine all calculated values and re-verify that each of the assumptions is met. Provide explicit justifications for each assumption with any numeric values listed clearly.

7. Select only the most appropriate test based on the thoroughly evaluated assumptions. For this test, list all of the parameters of the python function. Analyze these parameters and select the appropriate inputs that match the nature of our dataset and question. Ensure that none of the parameters have been automatically set to be true when they are not intended to be. Perform this selected test and provide test statistics and p-values to 3 decimal places. Do not stop until the test is complete.

**Advanced:**

I am analyzing variables related to hospital visits and demographics to answer the question, “Are there significant differences in length of stay across race categories?” The relevant variables are as follows:

- LOS (continuous): a patient’s length of stay in number of days
- RACE (categorical): “1” = white; “2” = black; “3” = hispanic; “4” = other

Embody the role of an experienced biostatistician and complete the following tasks:

1. Load the data, filtering only for relevant entries. Perform a data clean by removing NaN/blank entries in the relevant variables. Calculate the sample size for the cleaned and filtered dataset and store this value as a variable named sample_size. Calculate the size of each group, and store these as variables named {group}_size, where {group} is the name of each individual group.

2. Suggest the most relevant statistical method to use for analyzing this dataset, considering both the research question, nature of the data, and sample size. I believe the best choice would be ANOVA.

3. Rigorously list all of the critical assumptions that must be met in order to perform this statistical method.

4. Load the necessary libraries to perform the statistical functions and begin assessing these assumptions within the context of the dataset. Show all tables/figures/calculations necessary to assess the assumptions. Keep the following in mind:

- Do not perform any additional transformations on the data.

- If the test requires normality, assess this using histograms and Q-Q plots. Do not use the Shapiro-Wilk test. However, if the Central Limit Theorem is satisfied for each individual group (and not just the entire sample), no further testing for normality is required. Use {group}_size to assess this.

- If the test requires homoscedasticity, assess this using Levene’s test.

5. If any critical assumptions of the primary test are not met, identify a more appropriate alternative analysis. While some flexibility is allowed with less critical assumptions, ensure that the selected test provides valid and reliable results given the data characteristics. List the assumptions that are necessary for this alternative test, and based on your calculations determine if these have been met as well.

6. List the assumptions for the most appropriate test (list them again if the most appropriate test was the first one selected). Carefully examine all calculated values and re-verify that each of the assumptions is met. Provide explicit justifications for each assumption with any numeric values listed clearly.

7. Select only the most appropriate test based on the thoroughly evaluated assumptions. For this test, list all of the parameters of the python function. Analyze these parameters and select the appropriate inputs that match the nature of our dataset and question. Ensure that none of the parameters have been automatically set to be true when they are not intended to be. If you agree with my choice of ANOVA, perform this test and provide test statistics and p-values to 3 decimal places. Do not stop until the test is complete.

**KRUSKAL-WALLIS H**

**Basic:**

I am analyzing variables related to hospital visits and demographics to answer the question, “Are there significant differences in length of stay across race categories (White, Black, and Other only) for individuals who are 45 years old?” The relevant variables are as follows:

- LOS (continuous): a patient’s length of stay in number of days
- RACE (categorical): “1” = white; “2” = black; “3” = hispanic; “4” = other (exclude “3”)
- AGE (continuous): numerical age

Embody the role of an experienced biostatistician and complete the following tasks:

1. Suggest the most relevant statistical method to use for analyzing this dataset.

2. List and verify all of the critical assumptions that must be met in order to perform this statistical method.

3. If any critical assumptions of the primary test are not met, identify a more appropriate alternative analysis. If suggesting a new test, list and verify the assumptions for this new test.

4. Perform the most appropriate test and provide test statistics and p-values to 3 decimal places.

**Intermediate:**I am analyzing variables related to hospital visits and demographics to answer the question, “Are there significant differences in length of stay across race categories (White, Black, and Other only) for individuals who are 45 years old?” The relevant variables are as follows:

- LOS (continuous): a patient’s length of stay in number of days
- RACE (categorical): “1” = white; “2” = black; “3” = hispanic; “4” = other (exclude “3”)
- AGE (continuous): numerical age

Embody the role of an experienced biostatistician and complete the following tasks:

1. Load the data, filtering only for relevant entries. Perform a data clean by removing NaN/blank entries in the relevant variables. Calculate the sample size for the cleaned and filtered dataset and store this value as a variable named sample_size. Calculate the size of each group, and store these as variables named {group}_size, where {group} is the name of each individual group.

2. Suggest the most relevant statistical method to use for analyzing this dataset, considering both the research question, nature of the data, and sample size.

3. Rigorously list all of the critical assumptions that must be met in order to perform this statistical method.

4. Load the necessary libraries to perform the statistical functions and begin assessing these assumptions within the context of the dataset. Show all tables/figures/calculations necessary to assess the assumptions. Keep the following in mind:

- Do not perform any additional transformations on the data.

- If the test requires normality, assess this using histograms and Q-Q plots. Do not use the Shapiro-Wilk test. However, if the Central Limit Theorem is satisfied for each individual group (and not just the entire sample), no further testing for normality is required. Use {group}_size to assess this.

- If the test requires homoscedasticity, assess this using Levene’s test.

5. If any critical assumptions of the primary test are not met, identify a more appropriate alternative analysis. While some flexibility is allowed with less critical assumptions, ensure that the selected test provides valid and reliable results given the data characteristics. List the assumptions that are necessary for this alternative test, and based on your calculations determine if these have been met as well.

6. List the assumptions for the most appropriate test (list them again if the most appropriate test was the first one selected). Carefully examine all calculated values and re-verify that each of the assumptions is met. Provide explicit justifications for each assumption with any numeric values listed clearly.

7. Select only the most appropriate test based on the thoroughly evaluated assumptions. For this test, list all of the parameters of the python function. Analyze these parameters and select the appropriate inputs that match the nature of our dataset and question. Ensure that none of the parameters have been automatically set to be true when they are not intended to be. Perform this selected test and provide test statistics and p-values to 3 decimal places. Do not stop until the test is complete.

**Advanced:**

I am analyzing variables related to hospital visits and demographics to answer the question, “Are there significant differences in length of stay across race categories (White, Black, and Other only) for individuals who are 45 years old?” The relevant variables are as follows:

- LOS (continuous): a patient’s length of stay in number of days
- RACE (categorical): “1” = white; “2” = black; “3” = hispanic; “4” = other (exclude “3”)
- AGE (continuous): numerical age

Embody the role of an experienced biostatistician and complete the following tasks:

1. Load the data, filtering only for relevant entries. Perform a data clean by removing NaN/blank entries in the relevant variables. Calculate the sample size for the cleaned and filtered dataset and store this value as a variable named sample_size. Calculate the size of each group, and store these as variables named {group}_size, where {group} is the name of each individual group.

2. Suggest the most relevant statistical method to use for analyzing this dataset, considering both the research question, nature of the data, and sample size. I believe the best choice would be Kruskal-Wallis H.

3. Rigorously list all of the critical assumptions that must be met in order to perform this statistical method.

4. Load the necessary libraries to perform the statistical functions and begin assessing these assumptions within the context of the dataset. Show all tables/figures/calculations necessary to assess the assumptions. Keep the following in mind:

- Do not perform any additional transformations on the data.

- If the test requires normality, assess this using histograms and Q-Q plots. Do not use the Shapiro-Wilk test. However, if the Central Limit Theorem is satisfied for each individual group (and not just the entire sample), no further testing for normality is required. Use {group}_size to assess this.

- If the test requires homoscedasticity, assess this using Levene’s test.

5. If any critical assumptions of the primary test are not met, identify a more appropriate alternative analysis. While some flexibility is allowed with less critical assumptions, ensure that the selected test provides valid and reliable results given the data characteristics. List the assumptions that are necessary for this alternative test, and based on your calculations determine if these have been met as well.

6. List the assumptions for the most appropriate test (list them again if the most appropriate test was the first one selected). Carefully examine all calculated values and re-verify that each of the assumptions is met. Provide explicit justifications for each assumption with any numeric values listed clearly.

7. Select only the most appropriate test based on the thoroughly evaluated assumptions. For this test, list all of the parameters of the python function. Analyze these parameters and select the appropriate inputs that match the nature of our dataset and question. Ensure that none of the parameters have been automatically set to be true when they are not intended to be. If you agree with my choice of Kruskal-Wallis H, perform this test and provide test statistics and p-values to 3 decimal places. Do not stop until the test is complete.
