# Supplementary material for: ChatGPT for Univariate Statistics: Validation of AI-Assisted Data Analysis in Healthcare Research
Source: J Med Internet Res. 2025 Feb 7;27:e63550. doi: 10.2196/63550 (PMC11845875; doi:10.2196/63550)
Supplement: Multimedia Appendix 4 [file jmir_v27i1e63550_app4.docx]

**DATA TABULATION**

**Table S1.** Variable frequencies and proportions among standard cohorts and 45-year-old cohort.

|  | White | Black | Hispanic | Other | Total |
| --- | --- | --- | --- | --- | --- |
| All | 1638 (59.8%) | 593 (21.6%) | 235 (8.6%) | 274 (10.0%) | 2740 |
| Men | 962 (58.9%) | 350 (21.4%) | 153 (9.4%) | 169 (10.3%) | 1634 |
| Women | 676 (61.1%) | 243 (22.0%) | 82 (7.4%) | 105 (9.5%) | 1106 |
| 45-year-olds | 11 (40.7%) | 9 (33.3%) | 2 (7.4%) | 5 (18.5%) | 27 |
|  | White | Non-White* |  |  | Total |
| All | 1638 (59.8%) | 1102 (40.2%) |  |  | 2740 |
| Men | 962 (58.9%) | 672 (41.1%) |  |  | 1634 |
| Women | 676 (61.1%) | 430 (38.9%) |  |  | 1106 |
| 45-year-olds | 11 (40.7%) | 16 (59.3%) |  |  | 27 |

**Table S2.** Variable frequencies and proportions among age cohorts.

|  | 41–50 | 51–60 | 61–70 | Total |
| --- | --- | --- | --- | --- |
| All | 340 (12.4%) | 922 (33.7%) | 1478 (53.9%) | 2740 |
| Men | 234 (14.3%) | 569 (34.8%) | 831 (50.9%) | 1634 |
| Women | 106 (9.6%) | 353 (31.9%) | 647 (58.5%) | 1106 |

**DESCRIPTIVE STATISTICS**

**Table S3.** Descriptive statistics for patient age.

|  | Mean | Standard Deviation | Median | Interquartile Range |
| --- | --- | --- | --- | --- |
| All | 59.84 | 7.09 | 61 | 11.00 |
| Men | 59.28 | 7.19 | 61 | 10.00 |
| Women | 60.67 | 6.85 | 62 | 10.75 |

Age is in years.

**Table S4.** Descriptive statistics for patient length of stay.

|  | Mean | Standard Deviation | Median | Interquartile Range |
| --- | --- | --- | --- | --- |
| All | 9.63 | 9.08 | 7 | 9.00 |
| Men | 9.54 | 9.48 | 7 | 8.00 |
| Women | 9.77 | 8.46 | 7 | 9.00 |

Length of stay is in days.

**Table S5.** Descriptive statistics for patient total hospital charges.

|  | Mean | Standard Deviation | Median | Interquartile Range |
| --- | --- | --- | --- | --- |
| All | 135,242.03 | 97,396.23 | 104,682 | 133,814.00 |
| Men | 134,958.55 | 98,547.27 | 107,185 | 130,549.00 |
| Women | 135,663.73 | 95,701.80 | 103,492 | 139,391.00 |

Total charges are in USD ($).
