# Supplementary material for: ChatGPT for Univariate Statistics: Validation of AI-Assisted Data Analysis in Healthcare Research
Source: J Med Internet Res. 2025 Feb 7;27:e63550. doi: 10.2196/63550 (PMC11845875; doi:10.2196/63550)
Supplement: Multimedia Appendix 5 [file jmir_v27i1e63550_app5.docx]

**Figure S1.** Trial design flowchart for data management (data processing, categorization, and tabulation) and descriptive statistics. Gray boxes represent actions, green diamonds represent decisions based on ChatGPT’s outputs, and blue ovals represent coded responses.


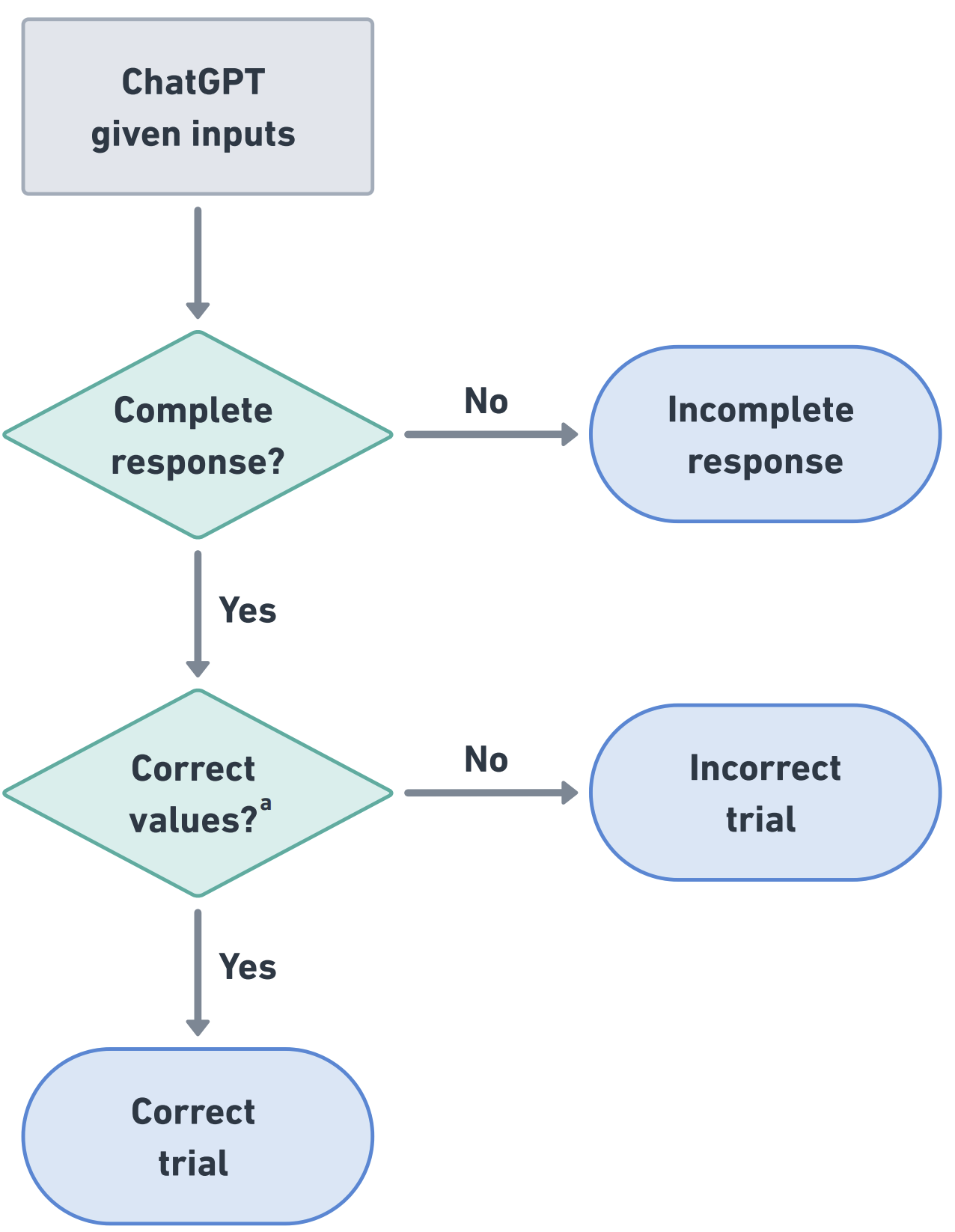


^a^The term “values” refers to frequencies (data processing and categorization trials), table entries (data tabulation trials), and statistical values (descriptive statistics trials).

**Figure S2.** Trial design flowchart for inferential statistics. Gray boxes represent actions, green diamonds represent decisions based on ChatGPT’s outputs, and blue ovals represent coded responses. Note that the outcomes for statistical assumptions and statistical values are independent.


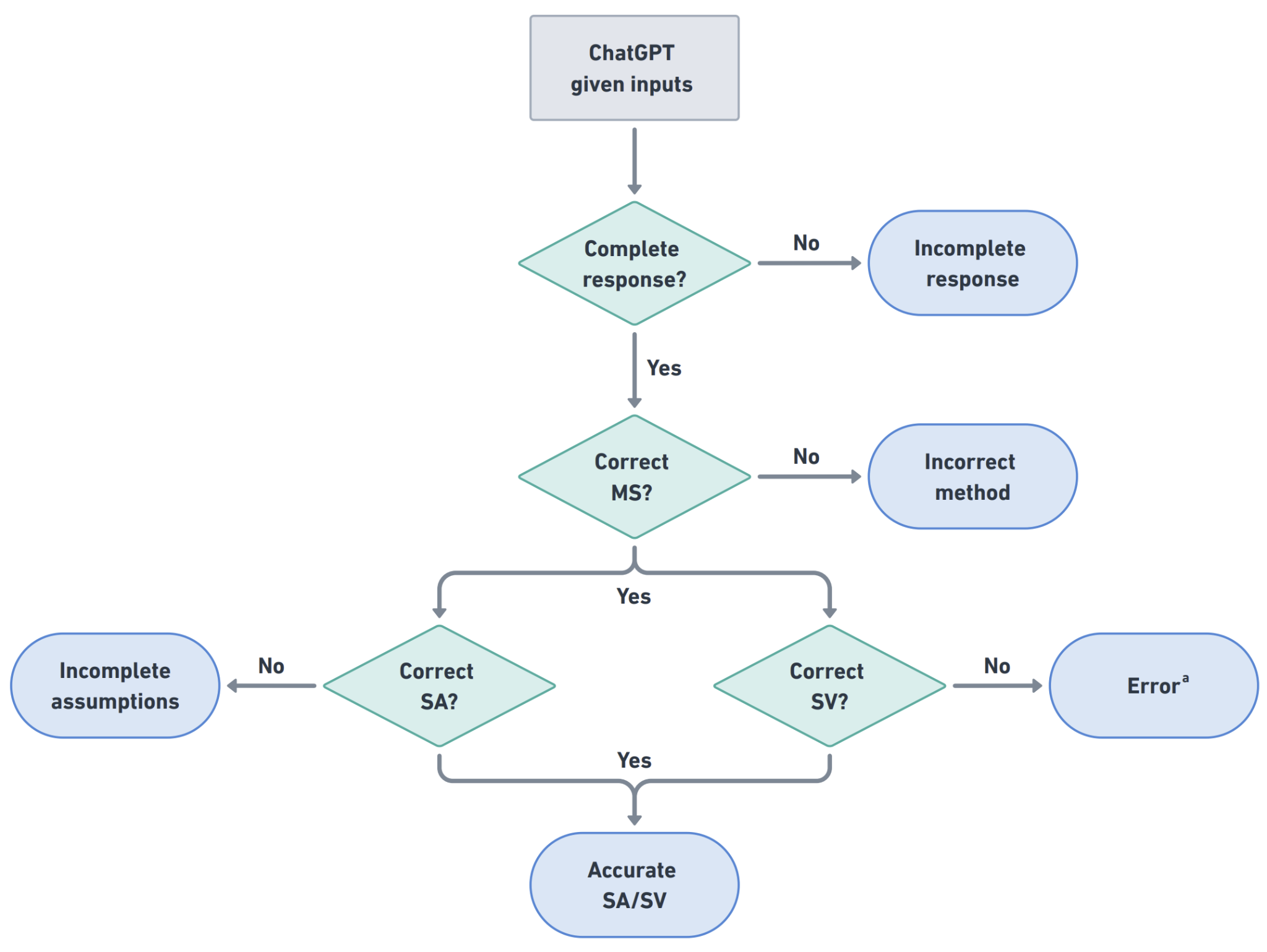


^a^Errors included coding errors, imputed values, and removed outliers.

MS = Method Selection; SA = Statistical Assumptions; SV = Statistical Values
